# The Head-Toes-Knees-Shoulders Revised: Links to Academic Outcomes and Measures of EF in Young Children

**DOI:** 10.3389/fpsyg.2021.721846

**Published:** 2021-09-07

**Authors:** Megan M. McClelland, Christopher R. Gonzales, Claire E. Cameron, G. John Geldhof, Ryan P. Bowles, Alexandra F. Nancarrow, Alexis Merculief, Alexis Tracy

**Affiliations:** ^1^Human Development and Family Sciences, Oregon State University, Corvallis, OR, United States; ^2^Learning and Instruction, University at Buffalo (SUNY), Buffalo, NY, United States; ^3^Center for Mind and Brain, University of California, Davis, Davis, CA, United States; ^4^Human Development and Family Studies, Michigan State University, East Lansing, MI, United States; ^5^Department of Education, University of York, York, United Kingdom

**Keywords:** self-regulation, executive function, school readiness, measurement, academic achievement

## Abstract

The measurement of self-regulation in young children has been a topic of great interest as researchers and practitioners work to help ensure that children have the skills they need to succeed as they start school. The present study examined how a revised version of a commonly used measure of behavioral self-regulation, the Head-Toes-Knees-Shoulders task (HTKS) called the HTKS-R, and measures of executive function (EF) was related to academic outcomes between preschool and kindergarten (ages 4–6years) in a diverse sample of children from families with low income participating in Head Start in the United States. Participants included 318 children (53% female; 76% White; and 20% Latino/Hispanic) from 64 classrooms in 18 Head Start preschools who were followed over four time points between the fall of preschool and the spring of kindergarten. Results indicated that children with higher HTKS-R scores had significantly higher math and literacy scores at all-time points between preschool and kindergarten. The HTKS-R was also a more consistent predictor of math and literacy than individual EF measures assessing inhibitory control, working memory, and task shifting. Parallel process growth models indicated that children who had high initial scores on the HTKS-R also had relatively higher initial scores on math and literacy. In addition, growth in children’s scores on the HTKS-R across the preschool and kindergarten years was related to growth in both children’s math and literacy scores over the same period independent of their starting points on either measure. For the HTKS-R and math, children’s initial scores were negatively associated with growth over the preschool and kindergarten years indicating that lower skilled children at the start of preschool started to catch up to their more skilled peers by the end of kindergarten.

## Introduction

Skills developed in early childhood lay the foundation for later success in school and life (Center on the Developing Child at Harvard University, 2011; McClelland et al., 2013). Meanwhile, many young children face difficulties as they move from early preschool and care settings to increasingly structured school environments during the transition to formal school settings (e.g., kindergarten in the United States; Gilliam and Shahar, 2006). In the search for sources of influence on school adjustment and success, children’s self-regulation has been identified as a malleable factor (Blair and Raver, 2015; Zelazo et al., 2016). A considerable body of evidence documents that self-regulation contributes to school success both prior to kindergarten and throughout formal schooling (Moffitt et al., 2011; McClelland et al., 2013; Blair and Raver, 2015). However, it remains a challenge to capture adequate variability in self-regulation measures in ways that are both ecologically valid and predict school readiness and success in young children.

Direct assessments of children’s self-regulation improve upon and complement traditional approaches requiring teacher or parent report (McClelland and Cameron, 2012). Considerable progress has been made in developing measures that capture young children’s self-regulation through a variety of approaches (Willoughby et al., 2012; Zelazo et al., 2013; Howard and Melhuish, 2017; Howard et al., 2019). Many assessments still require technology however (e.g., tablets or computers), are lengthy, expensive, or do not capture adequate variability in scores, especially for children from families with low income. Thus, validity and utility in applied settings and with diverse groups of children are less evident for most existing measures of self-regulation. In this study, we examine a revised version of the Head-Toes-Knees-Shoulders task (HTKS-R), a direct assessment of children’s behavioral self-regulation that requires multiple executive function (EF) components and has demonstrated strong reliability, validity, and predictive associations with various academic and classroom outcomes (McClelland et al., 2007a, 2014; Cameron Ponitz et al., 2009; Wanless et al., 2011; Schmitt et al., 2017; Lenes et al., 2020a; Gonzales et al., 2021). This study examines how well the HTKS-R and other measures of EF predict (a) variation in children’s academic outcomes in preschool and kindergarten and (b) growth in academic skills during the school transition.

### Self-Regulation and Executive Function

Self-regulation is a complex construct that includes a range of skills and is often defined differently according to discipline (e.g., developmental psychology, educational sciences, or cognitive psychology). Differences in conceptualizations reflect the wide variety of fields that examine self-regulation and the developmental and contextual framework in which self-regulation is considered (e.g., Blair, 2016). Scholars agree that self-regulation is a contextualized construct, consisting of controlling, directing, and planning to achieve social, academic, or personal goals or to avoid negative consequences (Baumeister and Vohs, 2004; Nigg, 2017; Bailey and Jones, 2019). In early childhood, research distinguishes among the self-regulation of emotions, cognitions, and behavioral outcomes, although there is considerable overlap among these domains (Blair and Raver, 2015; McClelland et al., 2015; Zelazo et al., 2016). Although self-regulation includes aspects of EF, it is also broader and captures other aspects of regulation including emotions and behavior (McClelland et al., 2015; Bailey and Jones, 2019).

In this study, we emphasize behavioral self-regulation, which refer to the use of executive function (EF) skills (i.e., complex working memory, complex response inhibition, and task shifting) in different situations, such as remembering to raise one’s hand and waiting to be called upon instead of shouting out an answer in class (McClelland et al., 2007b; Cameron Ponitz et al., 2008; Connor et al., 2010; Morrison et al., 2010). Our framework follows Miyake et al. (2000) and Garon et al. (2008) conceptualization of EF as showing both unity and diversity; that is, lower-order cognitive processes can be distinguished, but they are also all related to a higher-order skill. The ability to integrate multiple aspects of EF allows children to execute behaviors appropriate to the situation at hand. For example, in classroom settings, behavioral self-regulation is associated with remembering instructions, paying attention, and completing academic tasks (McClelland et al., 2007a; Cameron Ponitz et al., 2009). Self-regulation is also related to other constructs, such as effortful control, which stems from the temperament and personality literature and typically includes constructs, such as inhibitory control and attentional focusing but not working memory (McClelland et al., 2015). The area of study where EF and self-regulation meet brings together scholars from numerous disciplines, which is beneficial for theoretical and methodological diversity, but also the disadvantage of a proliferation of nomenclature (Morrison and Grammer, 2016; Morra et al., 2018). To address this issue, we define the constructs in our study in the context of existing work that is most applicable to our context of interest, which is early childhood learning environments. We also emphasize three of the most accepted underlying individual EF components in the unity-and-diversity conceptualization of EF, while acknowledging there is ongoing debate about components that space prevents us from comprehensively addressing here (Morra et al., 2018).

A large body of literature suggests that strong behavioral self-regulation is significantly associated with better achievement and social outcomes prior to and throughout children’s educational careers (McClelland et al., 2007a, 2013; Moffitt et al., 2011; Zelazo et al., 2016; Robson et al., 2020). In contrast, children who struggle with behaviors, such as talking out of turn and failing to complete assignments, have more difficulty in school (Ladd, 2003; McClelland et al., 2006).

### Self-Regulation and EF in Children From Families With Low Income

Socio-demographic risk due to membership in an oppressed cultural or socioeconomic status group increases children’s exposure to chronic stress and/or fewer opportunities to practice EF to regulate their behavior, which in turn influences their overall developmental trajectories (Blair and Raver, 2012; Ursache et al., 2016). Risk factors for children include coming from single-parent home, having parents with low educational attainment, and being from a minoritized race or ethnic group (Galindo and Fuller, 2010; Sektnan et al., 2010; Raver et al., 2012). These factors add pressure on children as they transition to formal school contexts—many of which perpetuate societal oppression rather than bolstering children’s nascent self-regulatory abilities (Love, 2019). In the United States, racial and ethnic minorities disproportionally experience the negative effects of systemic racism, including educational disparities and poverty (U.S. Census Bureau, 2013–2017). Together, research suggests that many children experiencing socio-demographic indicators of risk, which can make impulsivity key to thriving (Duran et al., 2020), have had few opportunities to practice EF prior to formal schooling and have difficulty transitioning to a more academic and EF-demanding classroom context (Blair and Raver, 2012; Blair, 2016). As a result, children from minoritized and otherwise oppressed groups are more likely to experience difficulty in school, report liking school less, and disengage from learning early in their academic careers (Blair and Raver, 2012; Roy and Raver, 2014). Meanwhile, interest in direct assessments of social-emotional learning, including behavioral self-regulation, is growing (McClelland et al., 2014; Halle and Darling-Churchill, 2016; Jones et al., 2016; Zelazo et al., 2016). Educational researchers and school leaders have a responsibility to use measures that can reliably and validly measure behavioral self-regulation in young children from diverse backgrounds (Harper, 2021).

### Measurement of Behavioral Self-Regulation With the HTKS

Structured self-regulation assessments involving direct observation of child responses during tasks have distinct advantages over the method of asking caregivers and teachers to report on children’s behavior. Direct assessments may be less prone to bias that researchers attribute to teachers’ beliefs and previous experiences with individual children (Loo and Rapport, 1998; Waterman et al., 2011). In addition, studies using both methods indicate that direct assessments of behavior provide different information than surveys (Gestsdottir et al., 2014).

The HTKS integrates multiple EF components into a game-like measure appropriate for children aged 4 to 8years (although the task has also been used with older adults; Cerino et al., 2018). Without needing any materials, the examiner relays several behavioral rules to the child, including: “touch your head,” “touch your toes,” “touch your shoulders,” and “touch your knees.” Children are first taught to “do the opposite” by touching their head when told to touch their toes and vice versa; new rules for these commands are added and changed as the task progresses in complexity. The task taps EF by requiring children to integrate multiple cognitive skills: (1) paying *attention* to instructions, (2) using *complex working memory* (Garon et al., 2008) to remember and execute new rules while processing the commands, (3) using *complex response inhibition* (Garon et al., 2008), specifically, *intentional motor inhibition* (Nigg, 2000), to inhibit their natural gross motor response that would follow each command while initiating the correct, unnatural, or “opposite” response, and (4) *task shifting* to switch their motor response when rules change (Morra et al., 2018).

The HTKS is moderately to strongly correlated with other established EF assessments and is a consistently strong indicator in latent variable models of EF (Allan and Lonigan, 2011; Schmitt et al., 2017). Because the HTKS has been shown to assess multiple aspects of EF, it also supports recent research supporting the greater unidimensionality of executive functions in relatively young children (Karr et al., 2018; Morra et al., 2018). Moreover, the task is short (5–7min) and easy to administer with good inter-rater reliability (*κ*=0.90; Cameron Ponitz et al., 2009; McClelland and Cameron, 2012), which makes it a practical tool for use in classrooms and across cultures and socioeconomic groups (Wanless et al., 2011; McClelland et al., 2014).

Accumulating research shows that the HTKS is one of the best-performing measures for predicting academic achievement in young children (Fuhs et al., 2014; Lipsey et al., 2017) and has strong construct and predictive validity (McClelland et al., 2007a, 2014; Cameron Ponitz et al., 2009; Cameron et al., 2019; Lenes et al., 2020b). Other research also indicates that the HTKS predicts academic achievement in diverse contexts and samples of children (Wanless et al., 2011; von Suchodoletz et al., 2013; Gestsdottir et al., 2014; McClelland et al., 2014; Cadima et al., 2015; Lenes et al., 2020b). Despite its general utility, the HTKS offers relatively less information about the behavioral self-regulation abilities of children with relatively low or nascent abilities; the HTKS exhibits floor effects among such populations. In the present study, we compare the HTKS-R to measures of EF that assess complex working memory, response inhibition, and set-shifting in their ability to predict academic outcomes in young children.

### Development of the HTKS-R

Although there is strong evidence to support the utility, reliability, and validity of the current three-part HTKS, there are also limitations to the task. For example, the gross motor demands of the task in addition to the cognitive complexity of the task may present challenges for young children, especially those facing socio-demographic risk factors. Studies have documented floor effects on the HTKS for children at socio-demographic risk, including children who are dual-language learners (DLLs; Caughy et al., 2013). This research indicates that the current HTKS does not adequately differentiate scores among the children that it is most important for schools to support—those facing disproportionate adversity. Thus, the HTKS-R, a revised version of the HTKS, was developed to address these issues (Gonzales et al., 2021).

The HTKS-R adds an additional section to the beginning of the task, which removes the motor and social demands inherent in the HTKS. Instead of requiring children to use gross motor movements, the first part of the HTKS-R asks children to *say* the opposite body part named by the examiner (head or toes) rather than having to *show* it. If children are successful, they proceed to the next parts of the task, which are essentially the same as the HTKS. Recent research has demonstrated that the HTKS-R displays stronger psychometric properties than the HTKS and showed greater variability in performance compared to the HTKS among young children from families with low income (Gonzales et al., 2021). Specifically, the HTKS-R showed floor effects for less than three percent overall in children between 48 and 60months of age, which was about 80% less than the floor effects on the HTKS. Moreover, the HTKS-R demonstrated construct validity and was more strongly related to other measures of EF and behavioral self-regulation across preschool and kindergarten than the HTKS (Gonzales et al., 2021). What remains unclear is how well the HTKS-R predicts (a) children’s academic outcomes in preschool and kindergarten compared to measures of EF and (b) how growth on the HTKS-R relates to growth in academic outcomes. Answering these questions was the goal of the present study.

### Current Study

The current study had two main research questions. First, we examined how the HTKS-R related to academic outcomes in relation to individual EF measures in young children from families with low income between the fall of preschool and the end of kindergarten. Based on research evaluating the HTKS (McClelland et al., 2007a, 2014; Cameron Ponitz et al., 2009; Wanless et al., 2011), we anticipated that children with high scores on the HTKS-R would have significantly higher academic achievement at all-time points and that the HTKS-R would be more consistently related to outcomes compared to individual measures of EF because the HTKS-R captures all aspects of EF in one assessment (McClelland et al., 2014).

The second research question examined how growth on the HTKS-R relates to growth in academic outcomes between preschool and kindergarten in children from families with low income. Based on previous research, we expected that children who showed greater growth over time on the HTKS-R would demonstrate similar growth in math and literacy skills (McClelland et al., 2014). We also hypothesized that children with low scores at the fall of preschool would show improvement in behavioral self-regulation, math, and literacy over the transition to kindergarten and would start to catch up to more skilled peers by the end of kindergarten (Montroy et al., 2016).

## Materials and Methods

### Participants

As part of a federally funded study to refine and evaluate the HTKS, 318 children (53% female) were recruited from 64 classrooms in 18 Head Start preschools in the Pacific Northwest over 2years. Participation in Head Start was used as a proxy for low-income status because this federally funded program is limited to children and families who meet poverty guidelines. Two cohorts were followed from fall of preschool (mean age=4.69years, *SD*=0.30) to spring of kindergarten (mean age=6.12years, *SD*=0.30). At fall of preschool, 15% of children were assessed in Spanish; at spring of kindergarten, 4% were assessed in Spanish. Parents received a demographic questionnaire and approximately 51% of forms were returned. Average primary caregiver education was 12.20years (*SD*=2.66), with 67% reporting a high school education or less. Participants were invited to report multiple racial/ethnic identities; 76% reported “White,” 20% reported “Latino/Hispanic,” and 4% marked another race/ethnicity. Of those who indicated “another race/ethnicity,” 26% marked two or more options, most frequently reporting Latino/Hispanic and White or African American and White.

### Procedure

The principal investigator and research team contacted preschool directors *via* telephone, e-mail, and in-person meetings to recruit local preschools using a convenience sampling approach. Graduate and undergraduate research assistants were trained on several measures of EF and academic achievement. Children were assessed in the fall and spring of preschool (Waves 1 and 2) and fall and spring of kindergarten (Waves 3 and 4) in their classroom or other school setting. Children provided verbal assent prior to each session, and sessions lasted 15–20min. When notified by a caregiver or teacher that a child spoke a language other than English, a bilingual assessor administered two subtests of the pre-language assessment screener (preLAS; Duncan and De Avila, 1998). Children whose home language was Spanish and received a score of 15 or more were administered all assessments in English; children who scored less than 15 points were assessed in Spanish. Children who spoke a language other than Spanish and did not pass the preLAS were not administered assessments at that time point. Spanish-speaking research assistants administered the preLAS at each wave of the study and children who received a preLAS were assessed by bilingual assessors at each time point.

### Measures

#### English Proficiency Screener

Two subtests of the preLAS were used to assess English language proficiency (Duncan and De Avila, 1998); “Simon Says,” which measures receptive vocabulary and “Art Show,” which measures expressive vocabulary. During the “Simon Says” subtest, assessors asked children to respond to verbal commands (e.g., “Simon says point to the door”). In the “Art Show” subtest, children were shown a picture book and asked to identify various items (Assessor: “What is this?” Child: “A cup.” Assessor: “What can you do with it?” Child: “Drink.”). Each subtest had 10 items, where children received 1 point for a correct response and 0 points for an incorrect response. If children scored 15 or more points they passed the preLAS and were assessed in English (Rainelli et al., 2017). Reliabilities ranged from *α*=0.77 to *α*=0.90 across the four time points.

#### Behavioral Self-Regulation Measure

##### Head-Toes-Knees-Shoulders Revised

The HTKS measured children’s behavioral self-regulation (Cameron Ponitz et al., 2008; McClelland et al., 2014). During the game, children were asked to do the opposite of what they were told (e.g., if told to touch their head, the child should touch their toes). The task increases in complexity until children were required to remember opposing rules involving four body parts (head, toes, knees, and shoulders). In an updated version of the HTKS, HTKS-R, an “Opposites” section was included at the start of the task. In this section, children were asked to *verbally* respond to prompts, e.g., “When I say toes, you say head.” Children received 2 points for a correct response, 1 point for a self-corrected response, and 0 points for an incorrect response. Scores ranged from 0 to 118, and the measure demonstrated good internal consistency: Wave 1 *α*=0.95, Wave 2 *α*=0.94, Wave 3 *α*=0.93, and Wave 4 *α*=0.92. Because this study took place in the context of developing a revision to the original HTKS measure, we considered an alternative version for a subset of children (*N*=128 in Wave 1 and *N*=100 in Wave 2) in which the new opposites section was administered after part 1 of the task, but only for children who scored below a cutoff on the first few practice items in part 1 (*N*=52 in Wave 1 and *N*=50 in Wave 2). Children who scored above the cutoff received full points for the opposites section. We ultimately did not adopt this approach (Gonzales et al., 2021). In the present study, we tested whether there were any differences in conclusions depending on the ordering; conclusions were not different, so we report results from the entire sample regardless of task ordering.

#### Executive Function Measures

##### Day-Night Stroop Task

The Day-Night Stroop task is a direct measure of complex response inhibition (Gerstadt et al., 1994). Children were shown a card with a picture of a sun or moon and were required to say the opposite of what they saw. For example, if shown a picture of a moon, a child should say “day.” Scores range from 0 to 32, where children received 2 points for a correct response, 1 point for a self-corrected response, and 0 points for an incorrect response. Reliability estimates for the present study were as: Wave 1 *α*=0.91, Wave 2 *α*=0.90, Wave 3 *α*=0.87, and Wave 4 *α*=0.83.

##### DCCS Task

The dimensional change card sort (DCCS) is a direct assessment that measures children’s task switching (Frye et al., 1995; Zelazo, 2006). During the task, children were asked to sort cards first by color, then by shape. If children received a score of 5 or more (out of 6) in phase one, children moved on to phase two where they were asked to sort cards differently depending on the presence or absence of a black border. The total score ranged from 0 to 24, where children received 1 point for a correct response and 0 points for an incorrect response. Reliability estimates for the present study were as: Wave 1 *α*=0.93, Wave 2 *α*=0.93, Wave 3 *α*=0.91, Wave 4 *α*=0.86.

##### Working Memory

Phonological working memory and semantic processing were assessed using the Woodcock Johnson-III or Woodcock-Munoz Batería III Auditory Working Memory task, a normed and standardized measure (Woodcock et al., 2001c; Muñoz-Sandoval et al., 2005). During the task, children were told a series of objects and numbers (e.g., two, 7, dog) and were asked to repeat back the objects first, then the numbers.

#### Academic Achievement

Academic achievement was measured using subtests of the Woodcock Johnson-III (WJ-III, Woodcock et al., 2001b). Age-normed *W* scores were utilized to represent total sum scores for the Applied Problems and Letter-Word WJ-III subtests (Mather and Woodcock, 2001). Higher *W* scores indicate better performance (i.e., more correct responses), and the *W* scale is especially suited for assessing growth (Najarian et al., 2019). Previous research has demonstrated high reliabilities (*α*<0.80) for all subtests (Woodcock et al., 2001a; Schrank et al., 2005). Testing on each subtest stops after six incorrect responses.

##### Applied Problems

Children’s mathematics skills were assessed using the Applied Problems subtest of the WJ-III (Woodcock et al., 2001a) or the Woodcock-Muñoz Batería III (Muñoz-Sandoval et al., 2005). The Applied Problems subtest measured children’s early mathematical operations (e.g., addition, subtraction, and counting). Children are shown a series of images and asked to quantify them, e.g., (“How many birds are there?”). As children progress through the measure, the items increase in complexity and children are asked to solve word problems, find the value of coins, and other more advanced mathematical operations (e.g., “What is the perimeter of this shape”). Children were given 1 point for a correct response and 0 points for an incorrect response.

##### Letter-Word Identification

Children’s literacy skills were measured using the Letter-Word Identification subtest of the WJ-III (Woodcock et al., 2001a) or the Woodcock-Muñoz Batería III (Muñoz-Sandoval et al., 2005). The Letter-Word subtest contained expressive and receptive items that capture letter identification and word-reading skills. Children are asked to name letters when shown a series of letters on a page, e.g., “Tell me the name of this letter” or when shown a list of words (e.g., the, on, and at), children are asked to read each word aloud. Children were given 1 point for a correct response and 0 points for an incorrect response.

### Analytic Approach

The analyses for research question 1 (RQ1) were conducted using Stata 16 (StataCorp, 2019), and the parallel process models for research question 2 were conducted in Mplus Version 8.4 (Muthén and Muthén, 2012). We examined whether it was necessary to account for the hierarchical structure in the data of children being nested within classrooms (Hox et al., 2017).

At Waves 1 and 2, there were five children per classroom on average; at Waves 3 and 4, there were two children per classroom on average because children moved from preschool (Waves 1 and 2) to kindergarten classrooms (Waves 3 and 4). Intraclass correlations (ICC) were calculated using the wave-specific classroom variable, and the following measures had ICCs greater than 0.10: at Wave 1, Woodcock Johnson Applied Problems subtest (0.12); at Wave 2, HTKS-R (0.12); at Wave 3, DCCS (0.12) and the Woodcock Johnson Applied Problems subtest (0.16); and at Wave 4, HTKS-R (0.20), and the Woodcock Johnson Applied Problems subtest (0.16). Thus, the analyses described below accounted for the nested structure of the data by utilizing clustered-robust standard errors using the wave-specific classroom as the cluster variable for RQ1 analyses and the cluster variable for Wave 1 (representing the preschool year classroom in the fall) for RQ2 analysis.

We examined missing data using logistic regression models to predict missingness on each variable. Missingness did not depend on any of the following demographics: age, gender, ELL status, parent education, parent marital status, and parent employment. Thus, to account for missingness, we ran models using a full information maximum likelihood (FIML) estimator within a structure equation model (SEM) framework. FIML uses all available data and generates less biased estimates compared to more traditional missing data methods like listwise or pairwise deletion (Enders, 2001).

### Primary Analyses

#### RQ1: Relations Between the HTKS-R and EF Measures and Academic Outcomes

We used within-time point path models to examine whether HTKS-R predicted academic achievement independent of the other EF measures: Day-Night, DCCS, and the Auditory Working Memory subtest of the Woodcock Johnson-III Tests of Achievement. We conducted these models within a SEM framework to utilize all available data instead of relying on listwise deletion in a regression framework.

#### RQ2: Growth in the HTKS-R and Growth in Academic Skills

We examined growth on children’s HTKS-R performance and measures of literacy and math skills from preschool through the end of kindergarten (Wave 1 to Wave 4) using a latent growth curve modeling approach. For each variable, we first fit a latent basis growth model estimating a latent intercept and slope parameter in an SEM framework from all available time points. The latent intercept parameter was measured by setting the factor loading for all-time points to 1. To allow for nonlinear development, the latent slope parameter was identified by setting the factor loading for children’s scores in the fall of preschool (Wave 1) to 0, spring of kindergarten (Wave 4) to 1, and allowing factor loadings for the spring of preschool (Wave 2) and fall of kindergarten (Wave 3) to be freely estimated^1^ (Grimm et al., 2016). In each model, we also constrained latent intercepts to 0 and residual variances to be equal over time. Model fit was also assessed *via* relative model fit for the linear growth model for each variable.

After fitting the growth models for each variable, we analyzed two parallel process models, one with literacy and the other with math, to investigate whether growth on the HTKS-R related to growth in academic outcomes. Parallel process models are used to determine whether change in one variable is related to change in another variable. The parallel process model and the unconditional linear growth models used random effects for the intercepts and slopes, and the intercept-slope covariance terms were freely estimated.

## Results

### Descriptive Statistics, Missing Data, and Attrition

Descriptive statistics for all variables are provided in Table 1. Children improved on behavioral self-regulation, EF tasks, literacy, and math at each wave, as expected. Missing data resulted mainly from attrition between waves but also occurred on specific tasks due to children refusing to complete a task or due to absences after three consecutive visits to the child’s classroom. Missing data due to children refusing to complete a task were typically very low (e.g., less than 2%). All other missing data were due to absences. Missing data not due to attrition were low except at spring of preschool (Wave 2) when there was 12–18% missing data on some direct measures. Specifically, rates of missing data at spring preschool were as: Day-night: 12%; DCCS: 14%; HTKS, 14%; Letter-Word Identification: 15%; Applied Problems: 13%; and Working Memory: 18%. Skewness and kurtosis values for the behavioral self-regulation, EF, and academic achievement tasks were within acceptable ranges (Kline, 2005), with skewness ranging from −2.45 to 0.95 and kurtosis ranging from 1.25 to 12.14. All models described below utilized clustered-robust standard errors to account for the nested nature of the data and heteroskedasticity.

**Table 1:** Descriptive statistics for all study variables.

Variable	Prekindergarten (Year 1)				Kindergarten (Year 2)			
	Fall (Wave 1)		Spring (Wave 2)		Fall (Wave 3)		Spring (Wave 4)	
	***N***	***M*** (***SD***)	***N***	***M*** (***SD***)	***N***	***M*** (***SD***)	***N***	***M*** (***SD***)
Age (months)	303	56.17 (3.63)	266	61.46 (3.66)	246	67.34 (3.68)	234	73.33 (3.61)
ELL (percent)	305	15.08%	278	10.07%	246	6.50%	235	3.83%
HTKS-R	296	36.71 (28.55)	236	52.75 (33.12)	246	71.96 (32.22)	235	87.57 (26.92)
Day-Night	297	19.84 (9.84)	241	22.91 (8.60)	245	26.17 (6.80)	234	28.50 (4.93)
Card Sort	290	11.04 (5.69)	233	13.39 (5.77)	243	15.30 (5.24)	235	17.26 (4.08)
WM	287	445.98 (10.14)	223	448.85 (14.65)	245	449.51 (17.62)	235	460.29 (19.84)
Literacy	294	317.42 (25.09)	232	328.69 (24.65)	246	343.68 (28.33)	235	382.83 (31.69)
Math	297	397.11 (24.26)	238	408.37 (24.01)	246	420.29 (22.41)	235	433.80 (20.35)

*ELL, English language learner status; HTKS-R, Head Toes Knees Shoulders – Revised; WM, Woodcock Johnson-III Tests of Achievement Auditory Working Memory subtest; Literacy, Woodcock Johnson-III Tests of Achievement Letter-Word Identification subtest; and Math, Woodcock Johnson-III Tests of Achievement Applied Problems subtest*.

### Research Q1: Results for the Relations Between the HTKS-R and EF Measures and Academic Outcomes

Within-time point correlations between all EF and academic measures are presented in Table 2. To address whether HTKS-R related to academic measures while controlling for other measures of EF, we conducted a series of within-time point path models predicting children’s literacy and math scores from their age, gender, and ELL status as well as their performance on the HTKS-R and EF measures: Card Sort, Day-Night, Working Memory. As shown in Table 3, children’s performance on the HTKS-R was the only variable that was a significant independent predictor after accounting for covariates when predicting children’s literacy and math scores at all four time points. Additionally, children who performed better on the Card Sort task had significantly higher literacy scores at the fall of kindergarten and higher math scores at the spring of preschool and fall of kindergarten. Children who performed better on the Day-Night task had significantly higher literacy scores at all-time points except the fall of kindergarten and significantly higher math scores at all-time points except the spring of preschool. The working memory task was only an independent predictor of literacy scores in the spring of kindergarten of math scores in the fall and spring of kindergarten. In each case, the full model with the HTKS-R accounted for an additional 9–13% of variance in children’s math scores and for an additional 2–7% of variance in children’s literacy scores compared to a model that excluded the HTKS-R.

**Table 2:** Within-timepoint Pairwise Correlations for EF and Academic Measures.

	Prekindergarten Fall Wave 1					Prekindergarten Spring Wave 2				
	1	2	3	4	5	1	2	3	4	5
HTKS-R	1					1				
Day-Night	0.53^**^	1				0.43^**^	1			
Card Sort	0.35^**^	0.22^**^	1			0.28^**^	0.18^*^	1		
WJWM	0.19^*^	0.17^*^	0.13^*^	1		0.36^**^	0.26^**^	0.19^*^	1	
Math	0.58^**^	0.38^**^	0.32^**^	0.32^**^	1	0.57^**^	0.38^**^	0.23^**^	0.25^**^	1
Literacy	0.32^**^	0.12^*^	0.29^**^	0.16^*^	0.33^**^	0.33^**^	0.30^**^	0.26^**^	0.23^**^	0.30^**^
	**Kindergarten Fall Wave 3**					**Kindergarten Spring Wave 4**				
	**1**	**2**	**3**	**4**	**5**	**1**	**2**	**3**	**4**	**5**
HTKS-R	1					1				
Day-Night	0.37^**^	1				0.37^**^	1			
Card Sort	0.48^**^	0.27^**^	1			0.38^**^	0.20^*^	1		
WJWM	0.34^**^	0.23^**^	0.22^**^	1		0.37^**^	0.27^**^	0.20^*^	1	
Math	0.63^**^	0.45^**^	0.42^**^	0.38^**^	1	0.58^**^	0.34^**^	0.31^**^	0.38^**^	1
Literacy	0.41^**^	0.31^**^	0.26^**^	0.18^*^	0.55^**^	0.45^**^	0.21^*^	0.26^**^	0.32^**^	0.52^**^

*HTKS-R, Head Toes Knees Shoulders – Revised. WJWM, Woodcock Johnson-III Tests of Achievement Working Memory subtest;*

* *p<0.05*;

** *p<0.01*;

^***^p<0.001.

**Table 3:** Within-timepoint Path Models Predicting Literacy and Math scores.

Variable	Prekindergarten (Year 1)		Kindergarten (Year 2)	
	Fall (Wave 1)	Spring (Wave 2)	Fall (Wave 3)	Spring (Wave 4)
**Literacy**				
Age (months)	0.08	0.09	0.09	0.18^**^
Gender	−0.03	−0.02	−0.01	−0.02
ELL status	−0.07	0.20^*^	−0.02	0.01
Card sort	−0.08	0.13	0.17^*^	0.01
Day-Night	0.18^**^	0.15^*^	0.05	0.08
WJWM	0.10	0.12	0.02	0.16^*^
HTKS-R	0.27^**^	0.19^*^	0.29^**^	0.32^**^
*ΔR* ^2^	0.04	0.02	0.05	0.07
*R*^2^ total	0.16	0.20	0.21	0.27
**Math**				
Age (months)	0.09^*^	0.06	−0.02	0.09^*^
Gender	0.04	0.05	0.01	−0.05
ELL status	−0.32^**^	−0.38^**^	−0.20^**^	−0.10^**^
Card sort	0.08	0.16^*^	0.20^**^	0.10
Day-Night	0.19^**^	0.08	0.13^*^	0.11^*^
WJWM	0.09	−0.02	0.12^*^	0.14^*^
HTKS-R	0.38^**^	0.40^**^	0.42^**^	0.43^**^
*ΔR* ^2^	0.09	0.10	0.12	0.13
*R*^2^ total	0.49	0.49	0.53	0.41

*Values represent β. ΔR^2^ calculated comparing the full model against a model without the HTKS-R. ELL status, English language learner status; WJWM, Woodcock Johnson-III Tests of Achievement Working Memory subtest; and HTKS-R, Head Toes Knees Shoulders – Revised*.

* *p<0.05*;

** *p<0.01*;

^***^p<0.001.

### Research Q2: Growth in HTKS-R and Growth in Academic Skills

To determine whether children’s growth on the HTKS-R related to their growth in academic outcomes, we first analyzed individual latent growth curve models of children’s performance on each of the HTKS-R and academic outcomes against an intercept only (i.e., a no-growth model) for each variable. In each case, absolute model fit was significantly improved in the latent growth curve model compared to the intercept only model. Relative and absolute model fit indices for the individual growth curve models for literacy, math, and the HTKS-R models are displayed in Table 4.

**Table 4:** Model fit statistics for linear growth models of individual variables.

	Literacy	Math	HTKS-R
CFI	0.998	0.988	0.920
TLI	0.998	0.988	0.920
AIC	9134.045	8692.231	9351.997
BIC	9164.015	8722.201	9381.966
RMSEA	0.017	0.043	0.138
Exact Model Fit	*χ*^2^ (6)=6.526, *p* =0.367	*χ*^2^ (6)=9.455 *p* =0.149	*χ*^2^ (6)=41.786 *p* <0.001
*χ*^2^ diff test^a^	*χ*^2^ (3)=49.87, *p* <0.001	*χ*^2^ (3)=24.53, *p* <0.001	*χ*^2^ (3)=53.91, *p* <0.001

a *Chi square difference test conducted against an intercept only (i.e., no-growth) model using procedures for an MLR estimator*.

**Figure 1:**
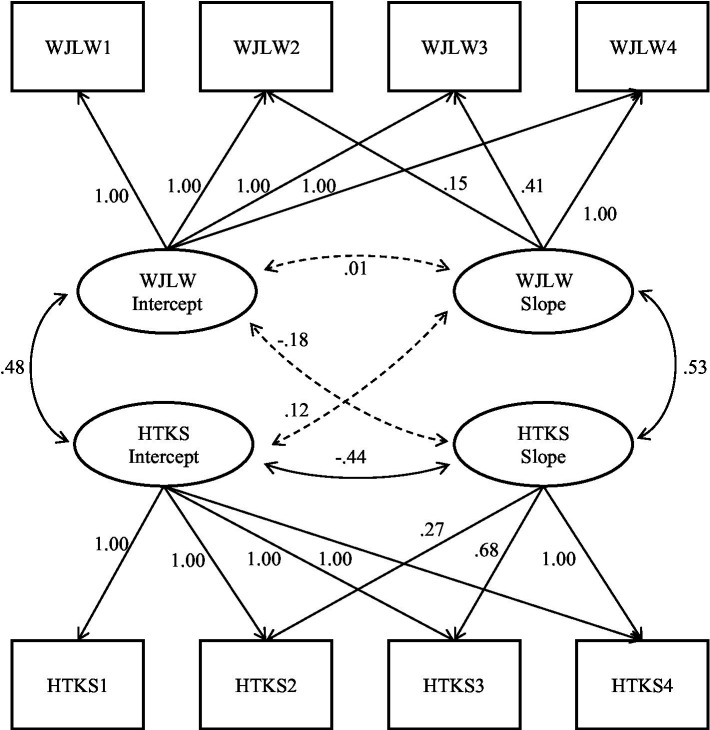
Literacy and HTKS-R parallel process model. Values represent unstandardized factor loadings and standardized covariances. Significant covariances and factor loadings at *p*<0.05 are displayed with solid lines. Non-significant loadings are displayed with dashed lines. Analyses revealed the following model fit indices: CFI=0.961; TLI=0.954; AIC=14363.732; BIC=14433.094; RMSEA=0.064; *χ*^2^(24)=47.342, *p*=0.003. WJLW=Woodcock Johnson Letter-Word subtest. HTKS-R, Head Toes Knees Shoulders – Revised.

**Figure 2:**
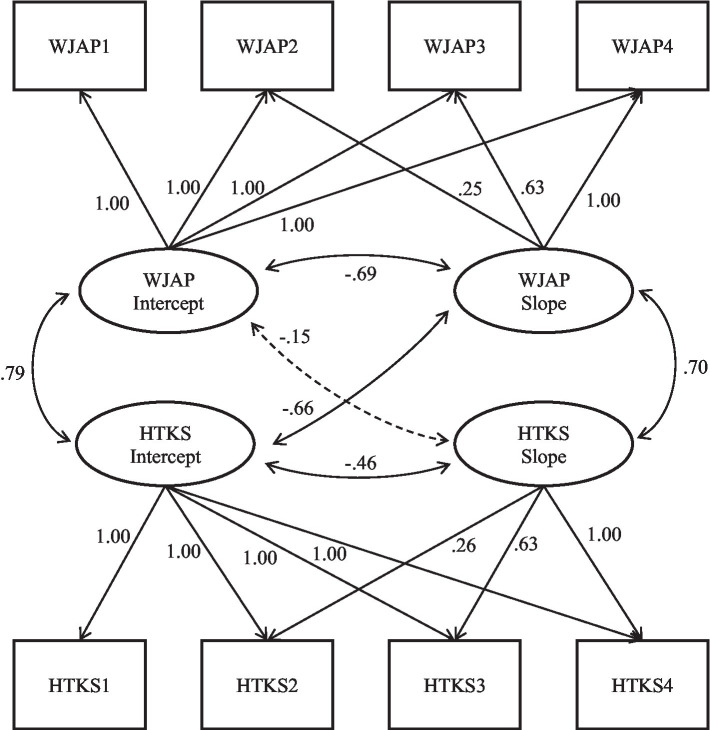
Math and HTKS-R parallel process model. Values represent unstandardized factor loadings and standardized covariances. Significant covariances and factor loadings at *p*<0.05 are displayed with solid lines. Non-significant are displayed with dashed lines. Analyses revealed the following model fit indices: CFI=0.971; TLI=0.966; AIC=13837.487; BIC=13906.848; RMSEA=0.067; *χ*^2^(24)=49.864, *p*=0.002. WJLW=Woodcock Johnson Letter-Word subtest. HTKS-R, Head Toes Knees Shoulders – Revised.

We next examined separate parallel process growth models to explore how the starting point (i.e., the intercept) and growth in performance (i.e., the slope) on the HTKS-R were related to the starting point and growth in children’s literacy skills (see Figure 1) as well as children’s math skills (see Figure 2). As shown in each figure, the HTKS-R intercept was significantly and positively related to the literacy and math intercepts. Therefore, high initial scores on the HTKS-R were associated with high initial scores on literacy and math. The HTKS-R intercept was also significantly negatively related to its own slope as well as the slope of math scores. That is, preschoolers who exhibited high HTKS-R scores at the fall of the preschool year tended to demonstrate slower increases in their scores on HTKS-R and math over the course of the preschool and kindergarten years, compared to children who had lower initial skill levels on the HTKS-R. This meant that children with lower skills at the start of preschool started to catch up to their more skilled peers by the end of the kindergarten year. Finally, the HTKS-R slope was significantly positively related to the slope of math scores and literacy scores, meaning that children who grew more on the HTKS-R grew more in both their math and literacy skills independent of their starting point on any of these assessments.

## Discussion

This study examined how children’s performance on a revised version of the HTKS structured observational measure of behavioral self-regulation, the HTKS-R, was related to their academic outcomes between the fall of preschool and the end of kindergarten, compared to other EF measures. We also examined how growth in the HTKS-R was related to growth on children’s math and literacy skills over this important transition to kindergarten (e.g., formal schooling in the United States). The HTKS has been shown to assess the cognitive aspects of EF (e.g., inhibitory control, working memory, and task shifting) in a single brief assessment (McClelland et al., 2014). Previous research has found that the HTKS is a significant predictor of children’s growth in early academic skills but young children placed at socio-demographic risk, including children who are DLLs, have been more likely to perform at lower levels on the HTKS (Caughy et al., 2013) and exhibit floor effects. Thus, the HTKS-R was developed to capture more variability in children’s nascent behavioral self-regulatory skills.

Results indicated that the HTKS-R was the most consistent predictor of children’s math and literacy scores compared to individual EF measures after accounting for covariates between the fall of preschool and spring of kindergarten. In addition, compared to children who had lower initial scores on the HTKS-R, children with high initial scores on the HTKS-R also had higher initial scores on literacy and mathematics but slower increases in scores on the HTKS-R and mathematics from preschool to kindergarten. Controlling for where they started, children who grew faster on the HTKS-R demonstrated faster growth in both their math and literacy skills.

### Relations Between the HTKS-R and EF Measures and Academic Outcomes

The present study found that the HTKS-R measure of children’s behavioral self-regulation was the strongest and most consistent independent predictor of both math and literacy skills when accounting for their performance on other individual measures of EF and socio-demographic covariates across the preschool and kindergarten years. A recent study of the measurement properties of the HTKS-R indicates that this revised version demonstrates significantly fewer floor effects than the HTKS during the preschool year and has stronger relations with other measures of EF (Gonzales et al., 2021). In the present study, correlations between the HTKS-R and other EF measures suggested that adding an initial section to the HTKS was most strongly related to the Day-Night measure of inhibitory control in the fall and spring of the prekindergarten year. Thus, it is possible that this first part of the task taps inhibitory control although in another recent study, the HTKS-R was also significantly related to a measure of task shifting and working memory in the fall and spring of the prekindergarten year (Gonzales et al., 2021). Future research should continue to examine how the HTKS-R is related to aspects of EF and self-regulation.

The present study extends this work to demonstrate consistently strong predictive relations between the HTKS-R and children’s early literacy and math skills in a sample of children attending Head Start (i.e., all families with low income). Although the sample was predominantly White (76%), which matched the demographic characteristics of the region, 20% of the sample self-identified as Latino/Hispanic and 15% of children were DLLs. Results of this study indicate that the HTKS-R captured variability in a sample of children placed at socio-demographic disadvantage. Furthermore, children’s performance on the HTKS-R also related to their academic outcomes similar to patterns found in other samples with a wider range of socioeconomic backgrounds (McClelland and Wanless, 2012; McClelland et al., 2014; Lenes et al., 2020b). Of note, the associations we observed between HTKS-R and academic outcomes were stronger than we have found in previous research using the HTKS (McClelland et al., 2014) and stronger than component measures of EF.

### Growth in HTKS-R and Growth in Academic Skills

Beyond documenting that HTKS-R performance is positively associated with early academic achievement in young children, results of the present study indicated that children who initially scored high on the HTKS-R also scored high on literacy and math measures at preschool entry. This result matches previous research showing that children’s concurrent levels of behavioral self-regulation and math and literacy are correlated (McClelland et al., 2007a; Schmitt et al., 2017) and may reflect the bidirectional coupling of these skills in early childhood (Schmitt et al., 2017; Cameron et al., 2019).

Moreover, children with high initial scores compared to their peers at the start of the preschool year showed slower increases in scores on the HTKS-R and math between preschool and kindergarten, compared to children with lower initial skill levels. This result suggests that children scoring lower at the start of preschool started to catch up to their more skilled peers by the end of the kindergarten year. Other research has shown this pattern with different samples of children (Montroy et al., 2016; Wanless et al., 2016), and the present study suggests that the HTKS-R can capture the variability in children’s scores over time. These results also suggest that children with high scores show less room to improve over time. We did not find evidence of ceiling effects on any measure, including the HTKS-R, so this result suggests a slowing of progress as opposed to measurement issues. Finally, growth on the HTKS-R was related to growth in math and literacy skills independent of children’s starting point on these assessments. This supports previous research finding that the slopes of behavioral self-regulation and math were correlated over time after children’s initial scores were taken into account (Cameron et al., 2019), although the present study also found that growth on the HTKS-R was related to growth in literacy skills.

Overall, results align with previous research supporting that growth in behavioral self-regulation is associated with growth in early academic skills and also support the HTKS-R as a measure that predicts growth in children’s early math and literacy skills between ages 4 and 6years. Behavioral self-regulation and EF are relevant for acquiring *new* skills for all children and especially for younger children and those from disadvantaged backgrounds who have had fewer opportunities to use EF as they practice self-regulating in different contexts (Blair and Raver, 2012; Ursache et al., 2016). Thus, it is not surprising that preschoolers who improved their performance on HTKS-R over time also improved in both mathematics and literacy skills. All children at the transition to formal schooling need working memory, task shifting, and inhibitory control as they work deliberately to recognize letters and letter sounds and apply phonological awareness as part of their burgeoning decoding skills (Cameron et al., 2012, 2015). After kindergarten, children start to automate these “building block” literacy skills and the HTKS-R and other measures that require EF are less strongly associated with these outcomes (Cameron, 2018).

### Practical Implications

There are a number of practical implications based on the results of the present study. First, the HTKS-R, like the HTKS, was developed as a short, easy-to-implement measure that captures aspects of EF (task shifting, inhibitory control, and working memory) in a single behavioral task. The HTKS and HTKS-R were developed as ecologically valid tasks that capture behavioral aspects of self-regulation also seen in classrooms and early learning settings (McClelland and Cameron, 2012). Results of this study and recent research suggest that, like the HTKS, the HTKS-R demonstrates construct validity (Gonzales et al., 2021) and predictive validity in the present study. Moreover, the HTKS-R is an improvement over the HTKS in reducing floor effects (Gonzales et al., 2021) and is a stronger predictor of academic outcomes in young children compared to previous research on the HTKS (McClelland et al., 2014). Practically speaking, this suggests that the HTKS-R can be reliably used with young children from families with low income with few floor effects, takes about 5minutes to administer, and significantly predicts early math and literacy skills. This lends support to using the HTKS-R as a kindergarten screening tool to identify children deserving of targeted support from professionals trained in strengths-based approaches and fostering behavioral self-regulation (e.g., classroom organization) as they make the transition into more formal school settings (Cameron and Morrison, 2011).

Another practical implication is the stronger predictive power found in this study in the HTKS-R overall measure of behavioral self-regulation compared to individual measures of EF. Using a single measure like the HTKS-R can be practically useful in school settings where teachers and other practitioners lack time, funds, or specialized materials to measure different aspects of EF separately. Although it is important in research settings to use multiple measures to adequately capture a complex construct like EF, research on the HTKS and the HTKS-R demonstrates that a single measure can perform similarly and in some cases, more strongly than individual measures of EF (McClelland et al., 2014; Lipsey et al., 2017).

### Limitations

The present study presented evidence supporting the predictive validity of the HTKS-R in a sample of children from families with low income, but there are a number of limitations to consider. First, although we controlled for demographic variables and baseline scores, we cannot infer causality from our analyses. Second, results of the present study are limited to children from families with low income participating in Head Start. This group of children was fairly diverse, with 20% families identifying as Latino/Hispanic, but was majority White (76%). Overall, the sample represented the demographics of the region. Other research using the HTKS-R in a separate sample of children in Head Start found similar relations with measures of EF, literacy, and math skills (McClelland et al., 2019). Moreover, previous research with the HTKS has found similar relations in a variety of socio-demographically and contextually diverse samples of children around the world (Wanless et al., 2011; von Suchodoletz et al., 2013; McClelland et al., 2014; Cadima et al., 2015; Cameron et al., 2019; Howard et al., 2019; Lenes et al., 2020b). However, caution should be taken in generalizing the results of the current study to other samples of children until research can be conducted in those groups with the HTKS-R. In sum, future research should further examine the final version of the HTKS-R with larger and more diverse samples of children and compare results across different samples of children.

## Conclusion

This study examined the predictive validity of the HTKS-R, which is a revised version of the widely used HTKS assessment of behavioral self-regulation. Results added to research on the construct validity of the HTKS-R (Gonzales et al., 2021) and demonstrate that children with higher scores on the HTKS-R had significantly higher math and literacy scores from preschool to kindergarten in a sample of children in the United States from families with low income. In addition, the HTKS-R more consistently predicted children’s early math and literacy skills compared to component measures of EF, and growth in HTKS-R scores across the transition to formal schooling was related to growth in math and literacy. Finally, we found that lower skilled children at the start of preschool started to catch up to their more skilled peers by the end of kindergarten in both behavioral self-regulation and math skills. These results suggest that the HTKS-R is a reliable and valid measure of behavioral self-regulation in young children, which predicts early school success in children from historically marginalized backgrounds.

## Data Availability Statement

The datasets presented in this article are not readily available because Oregon State University’s Internal Review Board does not allow the sharing of the data from this study. Requests to access the datasets should be directed to megan.mcclelland@oregonstate.edu.

## Ethics Statement

The studies involving human participants were reviewed and approved by the Oregon State University’s Internal Review Board. Written informed consent to participate in this study was provided by the participants’ legal guardian/next of kin.

## Author Contributions

MM lead efforts on conceptualizing, writing, reviewing results, and revising drafts. CG lead efforts on data analyzing and writing the results section. CC contributed to conceptualizing, writing, and reviewing drafts. JG and RB assisted with conceptualizing, data analyzing, and reviewing results and drafts. AN assisted with data analyses, methods, and reviewing drafts. AM and AT assisted with writing the methods and reviewing drafts. All authors contributed to the article and approved the submitted version.

## Funding

The research reported here was supported by the U.S. Department of Education Institute for Education Sciences grants # R305A150192 (PI: MM) to Oregon State University. The content is the responsibility of the authors and does not necessarily represent the official views of the Institute of Education Sciences or the U.S. Department of Education.

## Conflict of Interest

The authors declare that the research was conducted in the absence of any commercial or financial relationships that could be construed as a potential conflict of interest.

## Publisher’s Note

All claims expressed in this article are solely those of the authors and do not necessarily represent those of their affiliated organizations, or those of the publisher, the editors and the reviewers. Any product that may be evaluated in this article, or claim that may be made by its manufacturer, is not guaranteed or endorsed by the publisher.

## Footnotes

^1^The slope mean becomes the difference between expected values at times 1 and 4. The estimated factor loadings then represent the expected values at times 2 and 3 as fractions of the slope mean. This is a standard approach for the latent basis model (Grimm et al., 2016).
